# Impact of Ultrasound-Guided Suprascapular Nerve Block in Stroke Survivors With Hemiplegic Shoulder Pain Undergoing Neurorehabilitation: A Retrospective Case Series

**DOI:** 10.7759/cureus.69051

**Published:** 2024-09-10

**Authors:** Arvind K Sharma, Satyasheel S Asthana, Indrajit Deshmukh

**Affiliations:** 1 Physical Medicine and Rehabilitation, All India Institute of Medical Sciences, Raebareli, Raebareli, IND

**Keywords:** cerebrovascular accidents (cva), hemiparesis, hemiplegic shoulder, neurorehabilitation, physical medicine and rehabilitation, spadi score, stroke, suprascapular nerve block, ultrasound-guided nerve block, vas for pain

## Abstract

Background

Hemiplegic shoulder pain (HSP) is one of the most common complications seen in stroke survivors. HSP is an important cause of disability in these patients and may act as a barrier to rehabilitation and functional recovery. Suprascapular nerve block (SSNB) has been shown to be an effective treatment option for managing HSP, and it may also improve overall functional and motor recovery.

Methodology

This is a retrospective case series. Six stroke patients with HSP received an ultrasound-guided SSNB as a part of their inpatient individualized neurorehabilitation program. They were evaluated before the intervention and at 4 and 12 weeks of follow-up. Primary outcome measures were the Shoulder Pain and Disability Index (SPADI) score, active range of motion (AROM), and the visual analog scale (VAS) score of the hemiplegic shoulder. Secondary outcome measures were the passive range of motion (PROM) and manual muscle testing (MMT) of the hemiplegic shoulder.

Results

Of the six patients, four (66.7%) were male, four (66.7%) had hypertension, and two (33.3%) were also suffering from diabetes mellitus. Improvement was seen in the VAS score and the pain subscale of SPADI in all six cases at 12 weeks of follow-up. VAS score improvement was between 40% and 100%, while SPADI pain subscale score improvement ranged from 21.74% to 100%. Total SPADI score improved in all cases, with improvement ranging from 7.94% to 54.55%. No Improvement was seen in four of the six cases in the SPADI disability subscale. AROM showed an improvement in three of the six cases, with the most improvement in flexion (up to 55.56%). PROM improved in all six cases for flexion and abduction and in four cases for external rotation. MMT of only two patients improved by at least two grades.

Conclusions

SSNB is a safe and effective treatment option for patients with HSP. Along with an improvement in pain, the addition of SSNB in neurorehabilitation may play an important role in aiding functional and motor recovery in stroke survivors with HSP.

## Introduction

Stroke survivors suffer from various medical complications and hemiplegic shoulder pain (HSP) is one of the most common ones [1]. The incidence of HSP in stroke patients undergoing inpatient rehabilitation ranges between 24% and 64% [2]. HSP contributes to disability in stroke survivors and has also been linked to depression and a decreased quality of life. It may become a major obstacle in rehabilitation participation and the overall recovery of these patients [1].

The etiology of HSP is multifactorial and various pathologies have been linked to its development. These include factors such as impaired motor control and tone changes, soft tissue lesions, and various altered peripheral and central nervous activities [3]. Apart from central causes, nociceptive and peripheral neuropathic pain generation in the hemiplegic shoulder is also postulated as one of the possible mechanisms [4]. The loss of motor control has also been suggested to be an important causative factor in some studies [5]. The presence of severe HSP leads to worse motor outcomes in the affected upper limb [6].

About 70% of the sensory supply of the shoulder joint is provided by the suprascapular nerve. Suprascapular nerve block (SSNB) has proven to be an effective treatment option for shoulder pain in different degenerative shoulder pathologies [7]. A few studies have been conducted over the past two decades studying the efficacy and use of SSNB in HSP patients for pain and motor recovery, with promising results in terms of reduction of pain [8-13].

Pain is an important limiting factor when it comes to active participation in rehabilitation and patient compliance. Pain limits the use of the affected upper limb, thus contributing to the post-stroke limitation in performing activities of daily living. An improvement in pain may also allow better motor and functional recovery in this population of stroke survivors [14]. This case series examines the role of SSNB in improving pain and motor function of the affected upper limb in patients with HSP.

## Materials and methods

This was a single-center, retrospective case series. All six patients were treated in the inpatient neurorehabilitation unit of the Department of Physical Medicine and Rehabilitation at the All India Institute of Medical Sciences (AIIMS), Raebareli, Uttar Pradesh, India.

Inclusion and exclusion criteria

The inclusion criteria were age >18 years, diagnosed case of stroke with a radiological confirmation, and a visual analog scale (VAS) pain score of HSP of at least 30 mm. The exclusion criteria were more than one episode of stroke, prior injury/pathology of the affected shoulder, patients with uncontrolled diabetes mellitus, patients with cognitive problems, and patients with prior interventions for shoulder pain. A thorough baseline clinical evaluation and data collection was done for all six cases.

Intervention

Written and informed consent was obtained before the intervention. Local anesthetic sensitivity testing was done. The injection was administered with the patient in the sitting position under aseptic precautions. An ultrasound-guided SSNB was administered to all six cases. A linear ultrasound probe of frequency 5-12 MHz was used. An out-of-plane approach was used for administering the SSNB. A 90-mm long 22-G spinal needle was used. A mixture of a particulate corticosteroid (1 mL of triamcinolone acetonide 40 mg/mL), a local anesthetic (5 mL of injection bupivacaine 0.5%), and 0.9% normal saline (4 mL) was injected after ensuring appropriate needle placement using ultrasound guidance [8,9,11,15]. The patients were also prescribed an individualized neurorehabilitation program which included patient and caregiver education, postural care, activity modification, guidance regarding adequate nutrition, therapeutic exercises, and orthosis and assistive devices as required. Medical management of underlying diseases and/or comorbidities was continued.

Outcome measures

The primary outcome measures were the Shoulder Pain and Disability Index (SPADI) score; the active range of motion (AROM) of the hemiplegic shoulder in flexion, abduction, and external rotation; and the VAS score.

SPADI is a scale specifically designed for shoulder pathologies. Its internal consistency ranges between 0.8604 and 0.9507. It is a 13-item self-administered questionnaire with pain and disability subscales. Its test-retest reliability is high for both total and subscale scores and ranges between 0.6377 and 0.6552 [16].

VAS is a self-reported pain variable. The patient is asked to rate their perceived pain on a scale of 0-100 mm with 0 being “no pain” and 100 being “the worst imaginable pain.”

The secondary outcome measures were manual muscle testing (MMT) and passive range of motion (PROM) of the hemiplegic shoulder in flexion, abduction, and external rotation.

AROM and PROM were measured using a hand-held goniometer with the patient in a supine position. The range of motion (ROM) was recorded as degrees and then converted to percentages considering 0-180 degrees as the normal ROM for flexion and abduction, and 0-90 degrees for external rotation [17]. MMT was done for flexion, abduction, and external rotation of the hemiplegic shoulder using the Medical Research Council (MRC) grading which ranges from 0 to 5 [18].

Follow-up

After discharge, the patients were followed up at 4 and 12 weeks from the date of the intervention.

Statistical analysis

The data were analyzed using Microsoft Excel 365 (Microsoft Corp., Redmond, WA, USA). Quantitative data are expressed as mean with standard deviation along with the median. Qualitative data are shown as proportions and percentages. The changes in the primary and secondary outcomes are expressed as percentage changes in each individual case compared to the baseline.

## Results

The demographic details of the six patients are listed in Table 1.

**Table 1:** Demographic details of all six cases depicting age, gender, time since onset and type of stroke, and comorbidities.

Patient characteristics	Case 1	Case 2	Case 3	Case 4	Case 5	Case 6
Age (years)	60	49	47	71	67	51
Gender	Male	Female	Male	Male	Male	Female
Time since onset of stroke (in months)	17	32	6	8	6	1
Type of stroke (ischemic/hemorrhagic)	Hemorrhagic	Ischemic	Hemorrhagic	Ischemic	Ischemic	Ischemic
Hemiplegic side	Right	Left	Left	Right	Left	Left
Handedness	Right	Right	Right	Right	Right	Left
Hypertension	Yes	No	Yes	Yes	No	Yes
Diabetes mellitus	No	No	Yes	Yes	No	No

The mean age of the patients was 57.5 years (SD = 10.03 years). The average duration since the onset of stroke was 11.67 months (SD = 11.25 months). Of the six patients, four (66.7%) were male, four (66.7%) had hypertension, and two (33.3%) were suffering from diabetes mellitus. No post-intervention complications were observed in our study.

Improvement was seen in the VAS score and the pain subscale of SPADI in all six cases at 12 weeks of follow-up. VAS score improvement was between 40% and 100%, while SPADI pain subscale score improvement ranged from 21.74% to 100%. Total SPADI score improved in all cases, with improvement ranging from 7.94% to 54.55%. No Improvement was seen in four of the six cases in the SPADI disability subscale. AROM showed an improvement in three of the six cases, with the most improvement in flexion (up to 55.56%). PROM improved in all six cases for flexion and abduction and in four cases for external rotation. The changes in the VAS score, SPADI score, AROM, and PROM are depicted in Table 2.

**Table 2:** Changes in VAS, SPADI, AROM, and PROM in flexion, abduction, and external rotation of all cases at 4 and 12 weeks of follow-up. F0: baseline; F1: first follow-up at 4 weeks; F2: second follow-up at 12 weeks; VAS: visual analog scale; SPADI: Shoulder Pain and Disability Index; AROM: active range of motion; ROM: range of motion; PROM: passive range of motion

Outcome variable	Case 1	Case 2	Case 3	Case 4	Case 5	Case 6
VAS score						
Baseline (0–100 mm)	80.00	70.00	50.00	100.00	50.00	75.00
Percentage change F_1_-F_0_	100.00	42.86	20.00	50.00	70.00	46.67
Percentage change F_2_-F_0_	100.00	42.86	40.00	60.00	80.00	73.33
SPADI total						
Baseline (out of 130)	99.00	114.00	126.00	99.00	122.00	125.00
Percentage change F_1_-F_0_	25.25	7.89	3.17	38.38	13.11	25.60
Percentage change F_2_-F_0_	25.25	13.16	7.94	54.55	18.03	36.00
SPADI pain subscale						
Baseline (out of 50)	25.00	35.00	46.00	50.00	42.00	45.00
Percentage change F_1_-F_0_	100.00	11.43	8.70	58.00	38.10	46.67
Percentage change F_2_-F_0_	100.00	42.86	21.74	70.00	52.38	60.00
SPADI disability subscale						
Baseline (out of 80)	74.00	79.00	80.00	49.00	80.00	80.00
Percentage change F_1_-F_0_	0.00	0.00	0.00	18.37	0.00	13.75
Percentage change F_2_-F_0_	0.00	0.00	0.00	38.78	0.00	25.00
AROM flexion						
Baseline (% of normal ROM)	0.00	0.00	0.00	0.00	0.00	22.22
Percentage change F_1_-F_0_	0.00	0.00	0.00	44.44	11.11	27.78
Percentage change F_2_-F_0_	0.00	0.00	0.00	55.56	16.67	44.44
AROM abduction						
Baseline (% of normal ROM)	0.00	0.00	0.00	0.00	25.00	25.00
Percentage change F_1_-F_0_	0.00	8.33	0.00	22.22	2.78	25.00
Percentage change F_2_-F_0_	0.00	22.22	0.00	25.00	2.78	30.56
AROM ER						
Baseline (% of normal ROM)	0.00	0.00	0.00	0.00	0.00	0.00
Percentage change F_1_-F_0_	0.00	0.00	0.00	11.11	11.11	22.22
Percentage change F_2_-F_0_	22.22	0.00	0.00	22.22	22.22	22.22
PROM flexion						
Baseline (% of normal ROM)	61.11	55.56	50.00	33.33	66.67	44.44
Percentage change F_1_-F_0_	11.11	27.78	0.00	11.11	8.33	22.22
Percentage change F_2_-F_0_	22.22	27.78	5.56	22.22	16.67	22.22
PROM abduction						
Baseline (% of normal ROM)	50.00	55.56	50.00	25.00	38.89	44.44
Percentage change F_1_-F_0_	16.67	11.11	0.00	0.00	11.11	11.11
Percentage change F_2_-F_0_	25.00	19.44	5.56	2.78	16.67	16.67
PROM ER						
Baseline (% of normal ROM)	50.00	50.00	50.00	0.00	22.22	50.00
Percentage change F_1_-F_0_	0.00	5.56	0.00	11.11	11.11	0.00
Percentage change F_2_-F_0_	5.56	5.56	0.00	22.22	11.11	0.00

**Figure 1:**
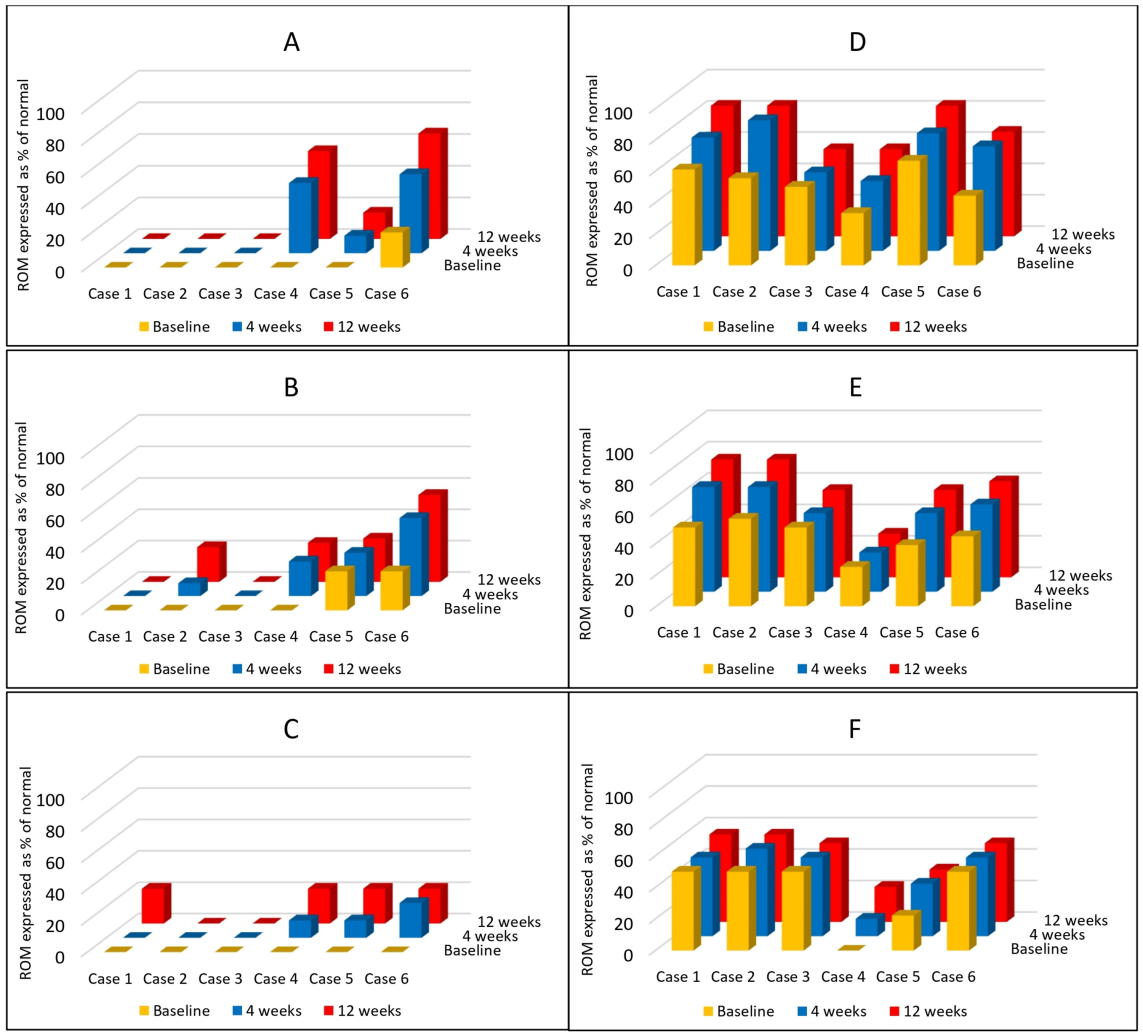
Changes in active and passive range of motion (ROM) (flexion, abduction, and external rotation) at 4 and 12 weeks. Panel A: changes in active ROM – flexion; B: changes in active ROM – abduction; C: changes in active ROM – external rotation; D: changes in passive ROM – flexion; E: changes in passive ROM – abduction; F: changes in passive ROM – external rotation. Cases 1-6 are expressed along the x-axis; ROM is expressed as percentages of the normal range along the y-axis, and time is expressed as the baseline, 4 weeks, and 12 weeks along the z-axis.

Changes in AROM and PROM at 4 and 12 weeks of follow-up are depicted in Figure 1.

**Figure 2:**
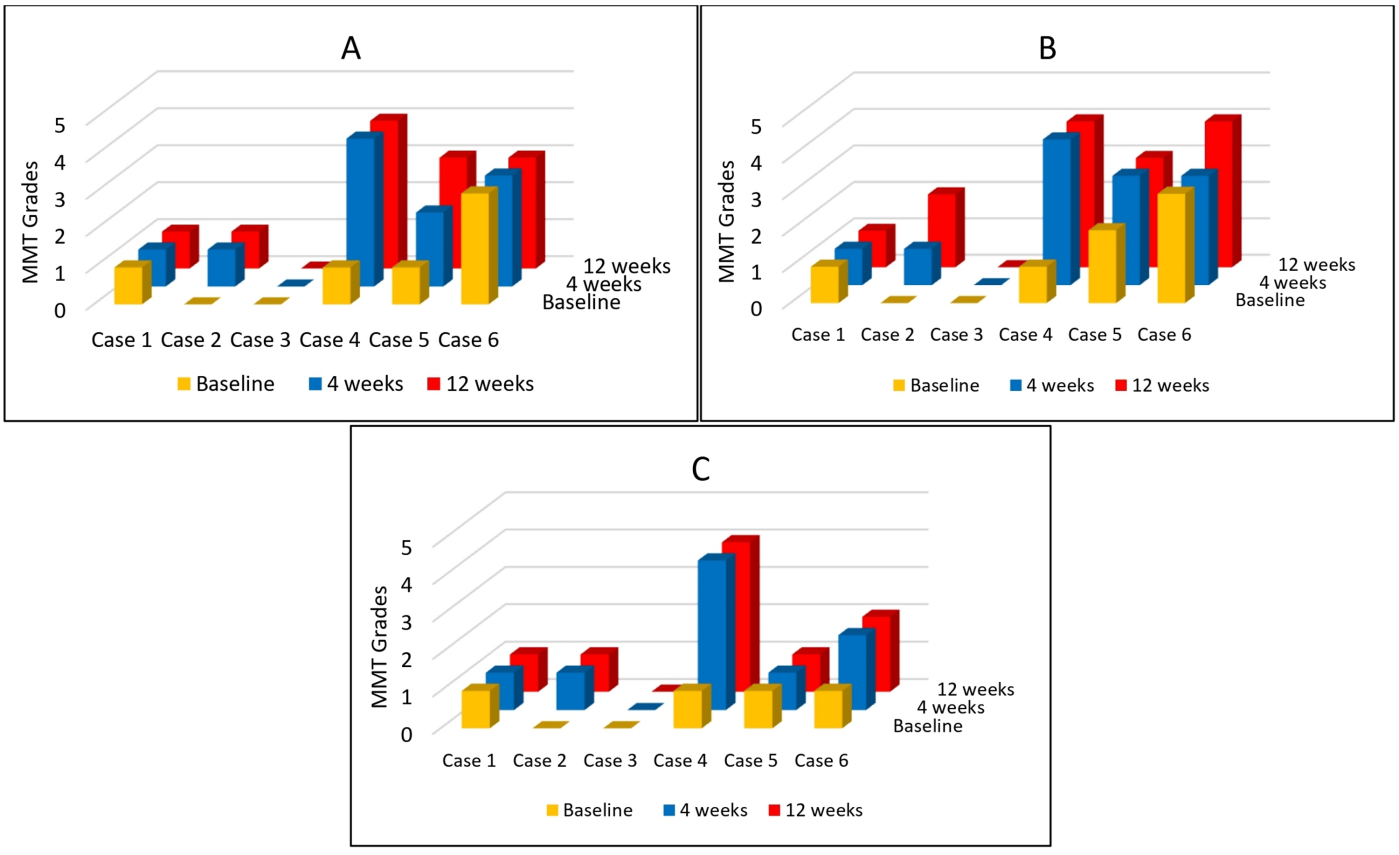
Changes in manual muscle testing (MMT) grades in flexion, abduction, and external rotation of cases 1-6 at 4 and 12 weeks of follow-up. A: changes in MMT grade of flexion; B: changes in MMT grade of abduction; C: changes in MMT grade of external rotation. Cases 1-6 are displayed along the x-axis; MMT grades are expressed along the y-axis (grades 0-5); time is expressed as the baseline, 4 weeks, and 12 weeks along the z-axis.

MMT grades were assessed for flexion, abduction, and external rotation of the affected shoulder. MMT of two patients improved by at least two grades. One of these two cases showed substantial improvement from grade 1 to grade 4 in all three movements. Two cases showed no improvement. The changes in MMT grades at 4 and 12 weeks of follow-up are depicted in Figure 2.

## Discussion

HSP is a collection of complex pathologies in the shoulders of stroke survivors. The biomechanics of the shoulder joint change due to HSP, which may be due to decreased motor control, secondary changes in surrounding soft tissues, glenohumeral joint subluxation, increased or decreased tone of muscle, and central sensitization [19]. HSP is usually managed by conservative or minimally invasive procedures. Conservative management includes a variety of pain medications (non-steroidal anti-inflammatory drugs, tricyclic antidepressants, opioids, or selective serotonin and norepinephrine reuptake inhibitors), anti-spastic drugs (baclofen), postural care, use of slings, therapeutic exercises, and various physical modalities. Minimally invasive procedures include SSNB, injection of botulinum toxin A in spastic muscles, radiofrequency ablation of the suprascapular nerve, intra-articular injections, and other soft tissue injections [19,20].

We presented six cases of stroke survivors with HSP in this series who were given an ultrasound-guided SSNB as a part of an inpatient neurorehabilitation program. The efficacy of SSNB for pain relief has been studied in the past. SSNB is much safer than intra-articular steroid injections [21,22]. Significant reduction in VAS scores was observed in many studies after SSNB at 4 weeks and 12 weeks from intervention [8,13,23]. The reduction of pain in this case series as assessed by the VAS score and the SPADI pain subscale score was along the lines of previous studies. Around 70% of the sensory innervation of the shoulder joint is provided by the suprascapular nerve. MRI findings similar to those of patients with adhesive capsulitis such as synovial capsule thickening, inflammatory processes in the synovium and the rotator cuff interval, and joint effusion have been seen in the painful hemiplegic shoulder [24]. A majority of these articular and peri-articular structures are innervated by the suprascapular nerve, which could explain the improvement in pain scores. SSNB temporarily decreases the pain perception for these patients by blocking the nociceptive inputs.

There is limited evidence regarding functional recovery in stroke survivors with HSP receiving SSNB. Functional recovery in this study was assessed using the disability subscale of the SPADI, along with changes in AROM and PROM. Although there was some improvement in the functional use of the affected upper limb in some cases, four of the six cases showed no change in the disability subscale score of SPADI at both follow-ups.

The motor function of the hemiplegic shoulder in our case series was assessed using AROM and MMT grades for flexion, abduction, and external rotation. AROM showed improvement in three of the six cases across all three movements, who were all patients with a stroke duration of less than one year. This improvement in AROM points to the usefulness of early intervention during the first year after stroke to prevent long-term morbidity. PROM improved in all six cases, which is similar to previous study findings [13]. The improvement in AROM as seen in some of the cases could be due to two reasons. First, it could be a result of the natural recovery of stroke due to neuroplasticity. Second, the reduction of pain enables the patient to participate in rehabilitation programs which may enhance the improvement in both PROM and AROM.

Due to HSP, the patient adapts to different movement behaviors. A posture or movement with minimal or no pain is adapted by the patient. Adapted movement behaviors, if persistent for a long time, can have long-term consequences such as contractures or physical deconditioning. Additionally, in stroke survivors, exercise-induced analgesia mechanisms may not work properly, and movement may increase pain, mainly in patients with central sensitization [25].

A significant association has been seen between poor arm motor function and the development of HSP [26]. The presence of HSP acts as a hindrance to the ability to perform activities of daily living. Thus, functional recovery is hampered, and the presence of pain further limits the patient’s participation in the rehabilitation program, contributing to poor recovery and further deterioration of motor function.

One of the major limitations of this case series was that it is a retrospective study with a small sample size of only six cases. There was no control group that was treated with a placebo or any other intervention.

## Conclusions

SSNB is a safe treatment option for stroke survivors with HSP. Early intervention for HSP during the first year after stroke could result in better outcomes in motor recovery in terms of pain, shoulder ROM, and muscle strength. Larger studies with an appropriate sample size need to be conducted in patients with HSP to fully understand the potential benefits of SSNB when administered as a part of an individualized neurorehabilitation program.

## References

[1] McLean DE (2004). Medical complications experienced by a cohort of stroke survivors during inpatient, tertiary-level stroke rehabilitation. Arch Phys Med Rehabil.

[2] Anwer S, Alghadir A (2020). Incidence, prevalence, and risk factors of hemiplegic shoulder pain: a systematic review. Int J Environ Res Public Health.

[3] Kalichman L, Ratmansky M (2011). Underlying pathology and associated factors of hemiplegic shoulder pain. Am J Phys Med Rehabil.

[4] Zeilig G, Rivel M, Weingarden H, Gaidoukov E, Defrin R (2013). Hemiplegic shoulder pain: evidence of a neuropathic origin. Pain.

[5] Kumar P (2019). Hemiplegic shoulder pain in people with stroke: present and the future. Pain Manag.

[6] Duray M, Baskan E (2020). The effects of hemiplegic shoulder pain on upper extremity motor function and proprioception. NeuroRehabilitation.

[7] Shanahan EM, Ahern M, Smith M, Wetherall M, Bresnihan B, FitzGerald O (2003). Suprascapular nerve block (using bupivacaine and methylprednisolone acetate) in chronic shoulder pain. Ann Rheum Dis.

[8] Picelli A, Bonazza S, Lobba D (2017). Suprascapular nerve block for the treatment of hemiplegic shoulder pain in patients with long-term chronic stroke: a pilot study. Neurol Sci.

[9] Adey-Wakeling Z, Crotty M, Shanahan EM (2013). Suprascapular nerve block for shoulder pain in the first year after stroke: a randomized controlled trial. Stroke.

[10] Allen ZA, Shanahan EM, Crotty M (2010). Does suprascapular nerve block reduce shoulder pain following stroke: a double-blind randomised controlled trial with masked outcome assessment. BMC Neurol.

[11] Terlemez R, Çiftçi S, Topaloglu M, Dogu B, Yilmaz F, Kuran B (2020). Suprascapular nerve block in hemiplegic shoulder pain: comparison of the effectiveness of placebo, local anesthetic, and corticosteroid injections-a randomized controlled study. Neurol Sci.

[12] Jeon WH, Park GW, Jeong HJ, Sim YJ (2014). The comparison of effects of suprascapular nerve block, intra-articular steroid injection, and a combination therapy on hemiplegic shoulder pain: pilot study. Ann Rehabil Med.

[13] Aydın T, Şen Eİ, Yardımcı MY, Kesiktaş FN, Öneş K, Paker N (2019). Efficacy of ultrasound-guided suprascapular nerve block treatment in patients with painful hemiplegic shoulder. Neurol Sci.

[14] Adey-Wakeling Z, Liu E, Crotty M, Leyden J, Kleinig T, Anderson CS, Newbury J (2016). Hemiplegic shoulder pain reduces quality of life after acute stroke: a prospective population-based study. Am J Phys Med Rehabil.

[15] Bensler S, Sutter R, Pfirrmann CW, Peterson CK (2018). Particulate versus non-particulate corticosteroids for transforaminal nerve root blocks: comparison of outcomes in 494 patients with lumbar radiculopathy. Eur Radiol.

[16] Roach KE, Budiman-Mak E, Songsiridej N, Lertratanakul Y (1991). Development of a shoulder pain and disability index. Arthritis Care Res.

[17] Barker KDD, Johnson MM (2021). The physiatric history and physical examination. Braddom’s Physical Medicine and Rehabilitation.

[18] Ciesla N, Dinglas V, Fan E, Kho M, Kuramoto J, Needham D (2011). Manual muscle testing: a method of measuring extremity muscle strength applied to critically ill patients. J Vis Exp.

[19] Bender L, McKenna K (2001). Hemiplegic shoulder pain: defining the problem and its management. Disabil Rehabil.

[20] Picelli A, Lobba D, Vendramin P (2018). A retrospective case series of ultrasound-guided suprascapular nerve pulsed radiofrequency treatment for hemiplegic shoulder pain in patients with chronic stroke. J Pain Res.

[21] Sun Y, Zhang P, Liu S, Li H, Jiang J, Chen S, Chen J (2017). Intra-articular steroid injection for frozen shoulder: a systematic review and meta-analysis of randomized controlled trials with trial sequential analysis. Am J Sports Med.

[22] Sencan S, Celenlioglu AE, Karadag-Saygı E, Midi İ, Gunduz OH (2019). Effects of fluoroscopy-guıded intraartıcular injectıon, suprascapular nerve block, and combınatıon therapy ın hemıplegıc shoulder paın: a prospective double-blınd, randomızed clınıcal study. Neurol Sci.

[23] Hou Y, Wang Y, Sun X, Lou Y, Yu Y, Zhang T (2021). Effectiveness of suprascapular nerve block in the treatment of hemiplegic shoulder pain: a systematic review and meta-analysis. Front Neurol.

[24] Távora DG, Gama RL, Bomfim RC, Nakayama M, Silva CE (2010). MRI findings in the painful hemiplegic shoulder. Clin Radiol.

[25] Hodges PW, Smeets RJ (2015). Interaction between pain, movement, and physical activity: short-term benefits, long-term consequences, and targets for treatment. Clin J Pain.

[26] Lindgren I, Jönsson AC, Norrving B, Lindgren A (2007). Shoulder pain after stroke: a prospective population-based study. Stroke.

